# Early Stages of Sea-Level Rise Lead To Decreased Salt Marsh Plant Diversity through Stronger Competition in Mediterranean-Climate Marshes

**DOI:** 10.1371/journal.pone.0169056

**Published:** 2017-01-19

**Authors:** Akana E. Noto, Jonathan B. Shurin

**Affiliations:** Section of Ecology, Behavior and Evolution, University of California, San Diego, La Jolla, California, United States of America; University of Sydney, AUSTRALIA

## Abstract

Climate change shuffles species ranges and creates novel interactions that may either buffer communities against climate change or exacerbate its effect. For instance, facilitation can become more prevalent in salt marshes under stressful conditions while competition is stronger in benign environments. Sea-level rise (SLR) is a consequence of climate change that affects the distribution of stress from inundation and salinity. To determine how interactions early in SLR are affected by changes in these two stressors in Mediterranean-climate marshes, we transplanted marsh turfs to lower elevations to simulate SLR and manipulated cover of the dominant plant species, *Salicornia pacifica* (formerly *Salicornia virginica*). We found that both *S*. *pacifica* and the subordinate species were affected by inundation treatments, and that subordinate species cover and diversity were lower at low elevations in the presence of *S*. *pacifica* than when it was removed. These results suggest that the competitive effect of *S*. *pacifica* on other plants is stronger at lower tidal elevations where we also found that salinity is reduced. As sea levels rise, stronger competition by the dominant plant will likely reduce diversity and cover of subordinate species, suggesting that stronger species interactions will exacerbate the effects of climate change on the plant community.

## Introduction

Climate change has altered ecological communities by shifting species ranges and interactions among co-occuring species. The impact of new or intensified interactions may be as important as the direct effects of climate on species [1,2]. For example, in rocky intertidal communities, the effect of a keystone predator becomes stronger as distributions of the immobile prey species are affected by climate change more than that of the predator [3]. Similarly, in alpine plant communities, a shift in plant distributions can lead to increased competition among plants that do not migrate effectively, thus exacerbating the effects of climate change [4]. In xeric environments, facilitative interactions that moderate desiccation stress become more prevalent with warming and decreased precipitation [5]. Thus, changes in species interactions can either exacerbate or mitigate the fitness effects of climate change depending on the circumstances.

In salt marshes, facilitative interactions among plant species often become more important in stressful environments [6,7]. The presence of neighboring plants increases fitness in the warmer, drier conditions of southern New England more than in cooler northern salt marshes [8]. Alternatively, climate change may lead to stronger competition if warming reduces stress associated with waterlogging, resulting in an increase in the competitive dominant and a decrease in diversity [9]. Finally, other experimental simulations of climate change had little effect on community interactions [10,11]. The shift in species interactions along climatic gradients therefore depends on the nature of physical forces and how they are mediated by different community members.

Sea-level rise (SLR) is one aspect of climate change with dramatic consequences in salt marshes. Tidal elevation determines salt marsh plant community structure [7,12] and SLR may therefore have large effects on communities. In salt marshes in Mediterranean climates, SLR could shift the balance between the two main stressors determining community composition: inundation and salinity [12–14]. SLR might lead to increased facilitation at low elevations as flood-tolerant plants benefit other species through sediment accretion and soil aeration [15,16]. Alternatively, SLR could lead to increased competition as inundation reduces salinity at mid-elevations by reducing evaporation [14] or physiological stress from inundation worsens the competitive ability of some plants. The effects of SLR on species interactions are likely to be different in marshes in Mediterranean climates compared to those on the east coast of the United States where a number of SLR studies have been done (e.g. [11,17]) and where wetter conditions make salinity stress less important.

To determine how species interactions vary with tidal elevation in salt marsh communities in Mediterranean climates, we conducted an experiment in which marsh turfs were transplanted to lower elevations in two southern California marshes to simulate SLR. While SLR is a gradual process and this experimental design resulted in abrupt change, we believe that this allows us to draw conclusions at least about the early stages of SLR. The tidal elevation treatment was crossed with manipulations of the density of the dominant plant species, *Salicornia pacifica*. We sought to determine whether interactions with the dominant plant became more competitive or more facilitative with SLR. We also asked how SLR and changes in interactions among plants would affect plant communities. This allowed us to determine how the impact of the dominant species on plant communities varies among situations representing different SLR scenarios.

## Materials and Methods

### Study system

This experiment was conducted at two marshes in San Diego County, CA. Kendall-Frost Mission Bay Marsh Reserve (KF) is a 16ha reserve located on a bay and surrounded by residential neighborhoods. Tijuana River National Estuarine Research Reserve (TJ) is 200ha, and although there are some nearby residential areas, it is within a larger reserve so that marsh areas are largely bordered by upland habitat. Permission to work in reserves was obtained from the California Department of Fish and Game as well as the University of California Natural Reserve System (KF), the Fish and Wildlife Service (TJ) and the National Estuarine Research Reserve System (TJ). The mean tidal range in this area is approximately 1.2m, but likely differs at the two sites. *Salicornia pacifica* (formerly *Salicornia virginica*) is the dominant plant species in mid-elevation marshes on the eastern coast of the Pacific from Baja California to Canada [18]. It is a perennial forb that grows upright, reproduces vegetatively and from seed, has a relatively broad elevation range and salinity tolerance [19], and is tolerant of inundation less than 18 hours per day [20]. Subordinate species in these marshes are mainly perennial forbs and include *Jaumea carnosa*, *Frankenia salina*, and *Batis maritima*.

### Experimental design

To determine the interactive effects of SLR and dominant species on salt marsh communities, we established an experiment in which marsh turfs were transplanted to lower elevations to increase the duration of tidal inundation. We selected plots of similar plant community composition within the mid- to high-marsh community, an area of high plant diversity, to transplant to the same elevation or one of two lower elevations. We then reduced cover of the dominant species, *S*. *pacifica*, by 0, 50 or 100% at each elevation to determine whether species interactions would be affected by SLR.

In December 2012, 0.5m x 0.5m plots were selected at KF and TJ. Plots were 1.7m and 1m above mean sea level at TJ and KF respectively, were similar in community composition and were inundated for similar amounts of time. Plots were transplanted 10cm lower or 30cm lower to represent conservative predictions of SLR in the next 50 and 100 years, respectively. We used conservative estimates as the eastern Pacific is expected to experience less SLR than other places [21–23]. These elevations resulted in turfs being moved to the lower limit of the *S*. *pacifica* range. We used real-time kinetic GPS and laser levels to measure elevations. We dug turfs to approximately 25-30cm, a depth which contained the majority of roots. We transplanted turfs to their starting elevations (to control for physical disturbance) or one of the two lower elevations such that their surface was flush with the ground. Plots were watered immediately after transplantation to reduce desiccation stress to plants.

**Fig 1:**
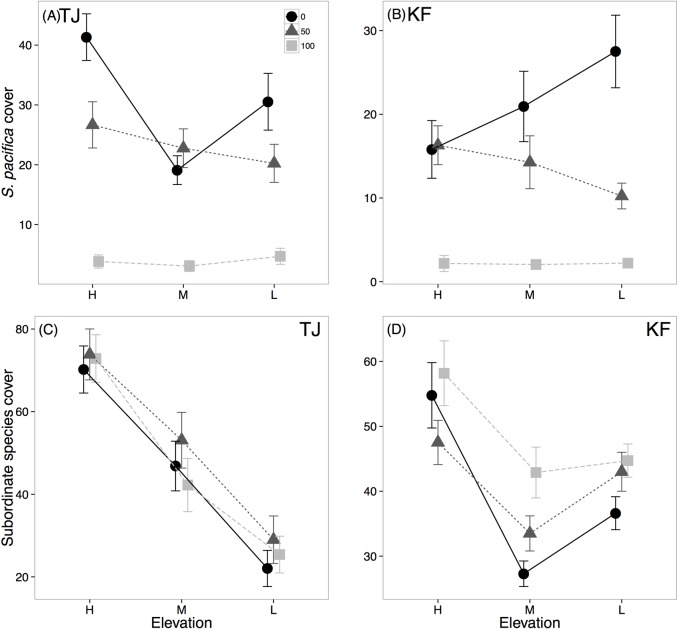
Percent cover of *S*. *pacifica* (top row) and subordinate species (bottom row) at high, medium and low elevations (left to right). Data are separated by *S*. *pacifica* removal treatment at TJ (A,C) or KF (B,D). Black circles indicate 0% *S*. *pacifica* removed, dark grey triangles indicate 50% and light grey squares indicate 100% removed.

After one month, we visually estimated percent cover and removed 0%, 50% or 100% of *S*. *pacifica* cover originally in each plot. Each site contained 5 replicates of each treatment resulting in 45 plots at each site (3x3x5). *S*. *pacifica* was removed by clipping at the soil surface, and any shoots growing back were removed throughout the experiment. Although this method removes aboveground biomass of the competitor while leaving belowground biomass intact, it has been used to assess strength of competition in other studies (e.g. [24,25]). In addition, very few shoots grew back into the plots, suggesting that clipped plants were unlikely to be competing strongly with remaining plants belowground (Fig 1A and 1B).

We sampled plots every March and October for three years. October is the time of peak biomass, and annual plants have emerged in March. At each sampling date we measured percent cover of every species. To determine how the treatments affected abiotic conditions, we measured soil salinity in each plot at each sampling date. We collected 2cm-diameter soil cores from the soil surface in the center of each plot and squeezed porewater through a Whatman number 3 qualitative grade filter onto a refractometer to measure soil salinity [26].

### Data analysis

We determined the effect of elevation and *S*. *pacifica* removal on the plant community using several different metrics. We measured percent cover of *S*. *pacifica* and all subordinate species, as well as Simpson’s diversity and richness of subordinate species. *S*. *pacifica* was excluded from calculations of plant diversity since it was experimentally manipulated. We used fixed effects models to determine how plant cover and diversity were affected by elevation, *S*. *pacifica* removal treatment, time and site. Because the amount of *S*. *pacifica* removed was variable among plots, we also used fixed effects models to test whether subordinate species cover at each elevation and site was related to observed *S*. *pacifica* cover rather than *S*. *pacifica* removal treatment. Data were square-root transformed when visual assessments revealed non-normally distributed residuals. *S*. *pacifica* cover and subordinate species diversity could not be transformed to meet assumptions, so we used randomizations of our data (1000 randomizations per test) to generate a null distribution to test hypotheses [27]. In the case of subordinate species diversity, analyses were done separately for each site. The same analyses were used to determine how soil salinity was affected by treatments.

Changes in plant community composition in response to the treatments were determined using distance-based redundancy analyses. We used Bray-Curtis distances and included interaction terms in hypothesis testing [28]. All analyses were done in R v. 3.2.0 [29]. Simpson’s diversity and redundancy analyses were done using the vegan package in R [30].

## Results

Elevation affected plant cover while the effect of *S*. *pacifica* removal was more variable. *S*. *pacifica* cover showed a significant removal X elevation X site interaction in which plots with unmanipulated *S*. *pacifica* cover differed significantly by elevation (Table 1; Fig 1A and 1B). Total cover of subordinate species (all other species) was significantly affected by an interaction between elevation, realized *S*. *pacifica* cover and site (F_2,468_ = 3.1594, p = 0.043; S1 Fig). Subordinate species cover was high when realized percent cover of *S*. *pacifica* was low, and at KF this effect was more pronounced at low elevations (S1 Fig). However, total cover of subordinate species was not affected by the level of *S*. *pacifica* removal (i.e. 0%, 50% or 100%; Table 1; Fig 1C and 1D). The two sites differed in their response to tidal height (Table 1), with subordinate cover uniformly decreasing as elevation decreased at TJ but with lowest subordinate cover at KF in middle elevation plots (Fig 1C and 1D).

**Table 1:** F-statistics from ANOVA and permutation tests of treatment effects.

	*S*. *pacifica* cover ^P^	Subordinate species cover	Salinity
Elevation (*E*)	--	62.3***	123.6***
Removal (*R*)	--	1.49	3.83*
Site (*S*)	--	0.39	323.3***
Time (*T*)	--	1.95^+^	67.6***
*E* × *R*	--	0.30	1.59
*E × S*	--	32.9***	32.9***
*R* × *S*	--	1.78	0.62
*E* × *T*	1.22	1.02	8.65***
*R* × *T*	2.24*	1.27	0.51
*S* × *T*	9.02***	4.14**	17.2***
*E* × *R* × *S*	4.73**	0.92	2.1^+^
*E* × *R* × *T*	0.40	0.26	0.63
*E* × *S* × *T*	1.19	1.35	3.79***
*R* × *S* × *T*	1.54^+^	0.22	0.33
*E* × *R* × *S* × *T*	0.49	0.25	0.68

Permutation tests cannot be done on terms included in significant interactions, and those were left blank.

^P^ Columns marked with a ^P^ were analyzed with permutation tests.

^+^P<0.1

*P<0.05

**P<0.01

***P<0.001.

**Fig 2:**
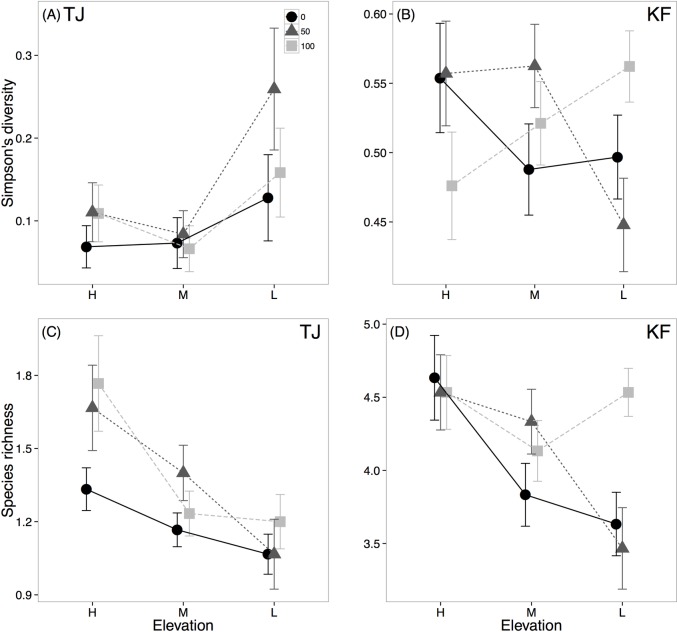
Simpson’s diversity (top row) and species richness (bottom row) of subordinate species diversity at high, medium and low elevations (left to right) at TJ (A,C) and KF (B,D). Data are separated by *S*. *pacifica* removal treatments (black circles indicate 0% *S*. *pacifica* removed, dark grey triangles indicate 50%, light grey squares indicating 100%).

Removal of *S*. *pacifica* also increased the diversity of the plant community (not including *S*. *pacifica*). This effect was strongest at low elevation and only occurred at KF (Table 2; Fig 2B). At KF, diversity increased at low elevations in plots from which all *S*. *pacifica* was removed. However, in plots still containing *S*. *pacifica* (i.e. 0% or 50% removal plots), diversity was reduced at low elevations (Fig 2). At TJ, diversity was affected by elevation but not *S*. *pacifica* removal (Table 2; Fig 2A). Species richness of subordinate species showed the same pattern as subordinate species Simpson’s diversity (Elevation X Removal: F_4,432_ = 2.93, p = 0.021; Fig 2C and 2D). Plant community composition at each site was significantly affected by elevation but not *S*. *pacifica* removal (S2 Fig).

**Table 2:** F-statistics from permutation tests of treatment effects on Simpson’s diversity.

	Subordinate species diversity	
	KF	TJ
Elevation (*E*)	--	5.20**
Removal (*R*)	--	1.56
Time (*T*)	--	0.40
*E* × *R*	--	0.66
*E* × *T*	--	0.45
*R* × *T*	--	0.53
*E* × *R* × *T*	1.63**	0.77

Permutation tests cannot be done on terms included in significant interactions, and those were left blank.

*P<0.05

**P<0.01

***P<0.001.

**Fig 3:**
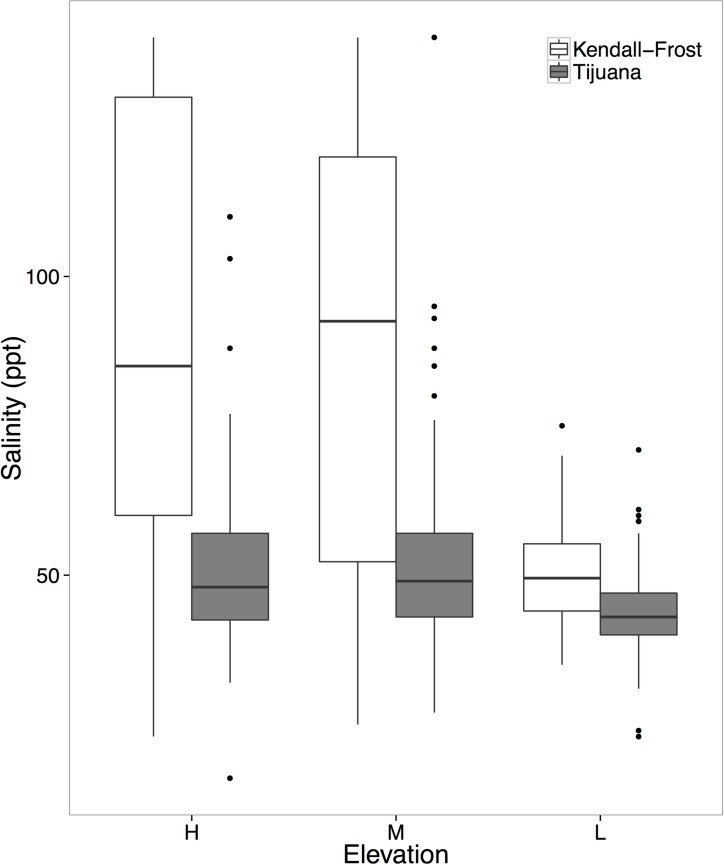
Salinity (parts per thousand) at Kendall-Frost (white) and Tijuana (gray) at high, medium and low elevation (left to right).

Salinity was significantly lower at low plots and in plots with no *S*. *pacifica* removal, although there was no interactive effect of removal and elevation (Table 1; Fig 3). The increase in salinity at high elevation was stronger at KF than at TJ (Table 1; Fig 3).

## Discussion

We found that experimentally simulated sea-level rise intensified the competitive effects of *S*. *pacifica* on subordinate plant species, indicating that early stages of sea-level rise are likely to favor stronger antagonistic interactions among salt marsh plant species. Removal of *S*. *pacifica* resulted in higher diversity of associated plants at KF, and this effect was strongest at the lowest tidal elevations. *S*. *pacifica* therefore competes more strongly with subordinate species at lower tidal elevations. This may be due to environmental changes at low elevations such as lower salinity or greater waterlogging stress and may ultimately lead to a decrease in subordinate species cover and diversity with SLR. Our results indicate that intensified competitive effects of the dominant species may magnify the effect of SLR on marsh plant community diversity.

Contrary to expectations, we found no indication that *S*. *pacifica* has a net facilitative effect on subordinate species at any tidal elevation (Fig 1), although its competitive effects were strongest at the lowest elevation. At KF, subordinate species diversity (Fig 2B and 2D) and subordinate species cover were most negatively affected by *S*. *pacifica* at low elevations (Fig 1D; S1 Fig). This shift to stronger competitive interactions with SLR was unexpected as facilitation often occurs at lower boundaries of distributions in salt marshes because these frequently inundated areas are stressful for plants [6]. *S*. *pacifica* is tolerant of flooding and low oxygen conditions [14,31], suggesting that it might be capable of facilitating other species low in the marsh through sediment accretion or soil aeration [31]. These facilitative benefits may take a long time to manifest, particularly in marshes dominated by *S*. *pacifica* rather than *Spartina alterniflora*, but the three-year duration of this experiment was likely sufficient to see minor effects as sediment accretion was measurable over the course of a year in marshes with similar community composition in San Francisco Bay, CA [32]. Nevertheless, we did not see evidence of facilitation at low elevations (Fig 1C and 1D), suggesting that in the early stages of SLR, facilitation is unlikely to rescue salt marsh plant communities from environmental change.

Our results suggest that salinity stress is an important structuring force in Mediterranean climate marshes, and it shows different patterns than in temperate marshes. Soil salinity was significantly lower at low tidal elevations (Fig 3), particularly at KF, and was consistently reduced when *S*. *pacifica* was not removed (Table 1). This suggests that *S*. *pacifica* facilitates subordinate species across all elevations due to its ability to reduce salinity via shading [26]. Simultaneously, *S*. *pacifica* competes with these species with the strongest competition at low elevations. Salinity in all plots was reduced at low elevations which leads to stronger competition in other marshes [6,8], suggesting that the shift in interaction strength we see here may also be due to a change in salinity stress across elevations. In fact, the competitive abilities of *S*. *pacifica* are likely to increase with decreasing salinity because salinity reduces its belowground biomass and branching [20]. The more pronounced salinity differences across elevations at KF compared to TJ (Fig 3) likely explain why a shift in competitive interactions only occurred at KF. Thus, in Mediterranean climates such as southern California, reduced salinity stress causes competition to be stronger in the low marsh. This is a notable contrast to trends observed on the east coast of the United States where higher rainfall typically leads to lower salinity stress in the high marsh [6].

Still, it is possible that the increase in competition at low elevations cannot be attributed solely to reduced salinity stress. Subordinate species benefitted from inundation in the absence of *S*. *pacifica* (Fig 2), but physiological stress from waterlogging at low elevations may be compounded by the presence of a competitor. However, several of the common subordinate species in this experiment (*Frankenia salina* and *Jaumea carnosa*) have been shown to be at least as tolerant of inundation as *S*. *pacifica* [33], suggesting that physiological stress is unlikely to drive competitive outcomes. In fact, inundation tolerance in subordinate species may help explain why facilitation was not observed in this case since facilitation that reduced inundation stress was likely less important for subordinate plant fitness than the detrimental effects of competition. At these conservative levels of SLR, the reduction in salinity stress seems to be a more relevant factor driving species interactions than inundation. As inundation stress increases, it may become a larger contributor to the direction and magnitude of species interactions [20,33].

It is important to note that while this experiment is intended to address the effects of SLR on plant communities via species interactions, SLR is a gradual process and abrupt changes to the community are an imperfect assessment of it. Many experiments that seek to investigate the effects of global change on ecological interactions necessarily share this problem (e.g. [4,34,35]). Similarly, SLR will have dramatic effects on marsh hydrology, and this study cannot address the effects of those changes on plant communities. However, similar results would be difficult to obtain by non-experimental methods, and we believe that these experiments provide valuable information about responses to global change despite this shortcoming.

Our results suggest that plant species diversity may decrease in the early stages of SLR as the strength of competition among plants increases. Changes in plant community composition and diversity may affect ecosystem services such as biomass production, habitat complexity and nitrogen accumulation [36]. This suggests that if the full suite of ecosystem services is to be maintained, efforts must be made to maintain species diversity, for example accounting for SLR and planting with high diversity when planning restoration projects.

We found that early stages of SLR cause a shift in the plant community by intensifying competitive effects of the dominant species on subordinates without a compensatory increase in facilitation. However, as SLR progresses, facilitation may eventually become stronger, suggesting that whether competitive or facilitative interactions dominate may depend on variation in environmental stress across time as well as space. In the early stages of SLR, these results suggest that tidal elevation has strong effects on species interactions via effects on salinity and eventually inundation, and large-scale environmental change can alter interactions locally. The effects of environmental change on species interactions depend on the type of environmental stress and its effects on different community members. Our results indicate that in the early stages of SLR, species interactions are likely to exacerbate the effects of SLR on salt marsh plant diversity in Mediterranean climates.

## Supporting Information

S1 Fig Subordinate species cover in relation to realized *S*. *pacifica* cover. Cover is shown at high (black), medium (green) and low (red) elevations at KF (top) and TJ (bottom). Solid lines indicate significant relationships while dotted lines indicate non-significance. Dashed lines indicate 95% confidence intervals.
(DOCX)

S2 Fig Distance-based redundancy analysis of plant community composition. Shown at TJ (left) and KF (right). Only species with scores >0.1 were included in this plot.
(DOCX)
