# Supplementary material for: Early Stages of Sea-Level Rise Lead To Decreased Salt Marsh Plant Diversity through Stronger Competition in Mediterranean-Climate Marshes
Source: PLoS One. 2017 Jan 19;12(1):e0169056. doi: 10.1371/journal.pone.0169056 (PMC5245857; doi:10.1371/journal.pone.0169056)

**S1 Fig. Subordinate species cover in relation to realized *S. pacifica* cover**. Cover is shown at high (black), medium (green) and low (red) elevations at KF (top) and TJ (bottom). Solid lines indicate significant relationships while dotted lines indicate non-significance. Dashed lines indicate 95% confidence intervals.


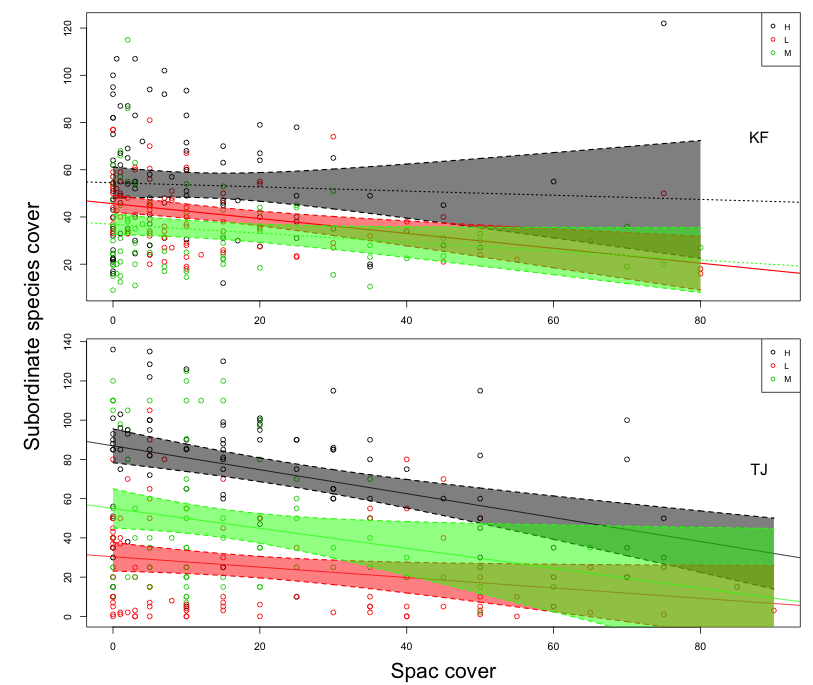

Supplement: S1 Fig — Cover is shown at high (black), medium (green) and low (red) elevations at KF (top) and TJ (bottom). Solid lines indicate significant relationships while dotted lines indicate non-significance. Dashed lines indicate 95% confidence intervals. (DOCX) [file pone.0169056.s001.docx]
