# Supplementary material for: Early Stages of Sea-Level Rise Lead To Decreased Salt Marsh Plant Diversity through Stronger Competition in Mediterranean-Climate Marshes
Source: PLoS One. 2017 Jan 19;12(1):e0169056. doi: 10.1371/journal.pone.0169056 (PMC5245857; doi:10.1371/journal.pone.0169056)

**S2 Fig. Distance-based redundancy analysis of plant community composition**. Shown at TJ (left) and KF (right). Only species with scores >0.1 were included in this plot.


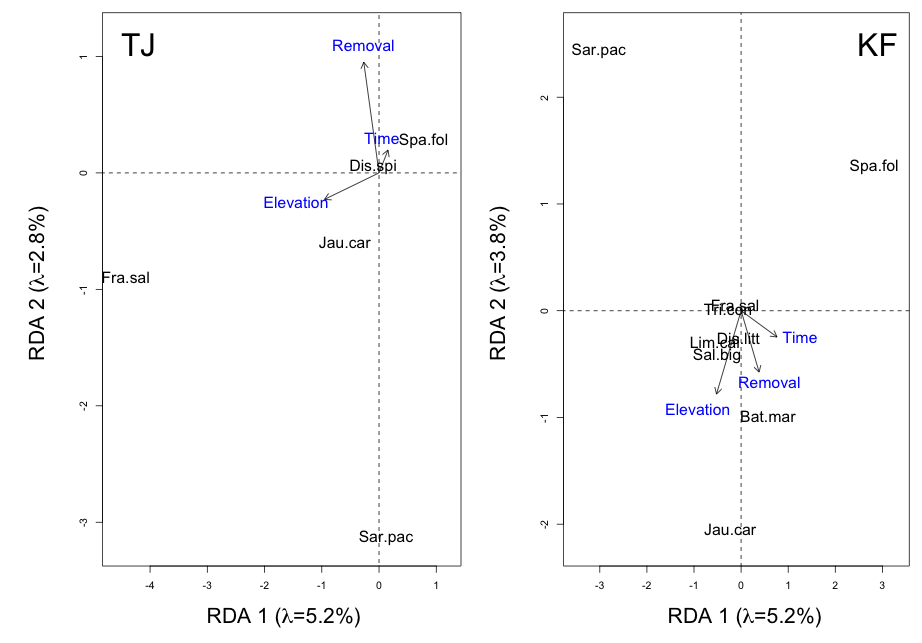

Supplement: S2 Fig — Shown at TJ (left) and KF (right). Only species with scores >0.1 were included in this plot. (DOCX) [file pone.0169056.s002.docx]
